# Effect of exposure to extremely low frequency magnetic fields on melatonin levels in calves is seasonally dependent

**DOI:** 10.1038/srep14206

**Published:** 2015-09-18

**Authors:** Tereza Kolbabová, E. Pascal Malkemper, Luděk Bartoš, Jacques Vanderstraeten, Marek Turčáni, Hynek Burda

**Affiliations:** 1 Department of Game Management and Wildlife Biology, Faculty of Forestry and Wood Sciences, Czech University of Life Sciences Prague, Kamýcká 129, 165 21 Prague 6, Czech Republic; 2 Department of General Zoology, Faculty of Biology, University of Duisburg-Essen, Universitätsstr. 2, 45117 Essen, Germany; 3 Department of Ethology, Institute of Animal Science, Přátelství 815, 104 00 Prague-Uhříněves, Czech Republic; 4 Research Center on Environmental Health and Work Health, School of Public Health, Université Libre de Bruxelles, CP 593, Route de Lennik 808, 1070 Brussels, Belgium

## Abstract

The question of health effects of extremely low frequency (50/60 Hz) magnetic fields (ELFMF) has been widely discussed, but the mechanisms of interaction of these fields with biological systems for intensities relevant to human and animal exposure are still under question. The melatonin (MLT) hypothesis suggests that exposure to ELFMF might decrease MLT production thereby promoting cancerogenesis. So far, most studies of MLT secretion under exposure to ELFMF reported negative or inconsistent results. Here, we measured salivary MLT in 1–2 months old cattle calves exposed to 50 Hz-MF in the hundreds of nT-range. We found an inhibitory effect of the ELFMF upon MLT secretion in winter (in accordance with the MLT hypothesis). In contrast, in summer, MLT concentration was increased by ELFMF exposure (contrary to the MLT hypothesis). The inhibitory effect in winter was much stronger than the positive effect in summer. We hypothesize that this season-dependent effect upon MLT synthesis might by mediated by an effect of ELFMF upon the serotonin metabolism and conclude that future tests of ELFMF effects should also measure serotonin levels and consider association with the seasonal effects (photoperiod or temperature) during the exposure.

The question of health effects of extremely low frequency magnetic fields (ELFMF) has been widely discussed, but the mechanisms of interaction of these fields with biological systems for intensities relevant to human and animal exposure are still under question. Several mechanisms of interaction of ELF-MFs with biological systems have been suggested: among others, magnetite interactions, radical pair mechanism, activation of voltage-gated calcium channels, ion cyclotron resonance interactions with electric field fluctuations in the cell membrane (cf.^1^,^2^,^3^,^4^).

Influence on spatial memory in rodents as well as increased risk of childhood leukemia have been, however, widely documented^5^,^6^,^7^,^8^,^9^. The “melatonin hypothesis”^10^ suggested that exposure to ELFMF decreases melatonin (MLT) production and, since MLT has cancerostatic properties, thus might promote development of breast cancer in humans. More recently, it has been suggested that ELFMF might indirectly promote cancer by affecting the circadian clock function of retinal cryptochromes^11^,^12^, which regulates the phototransmission to the suprachiasmatic nucleus (considered the master biological clock). Since MLT is the main hormonal biomarker of circadian biorhythms^13^, study of its secretion under exposure to ELFMF is relevant also with respect to the latter hypothesis. Related data from the literature (reviewed in^8^) are, however, contradictory. About half (46%) of 43 studies on mammals found a relationship between ELFMF and MLT secretion (decrease in 17 cases and increase in 3 cases), while the other half (54%) failed to show any effect or the authors concluded that there was no effect because their experiments provided inconsistent or contradictory results^7^. It is, however, difficult to generalize these results or to explain deviating findings. First, the species differed. Around 75% of the studies were done on laboratory rats, mice and *Phodopus* hamsters, while only 25% were using larger mammals such as cattle, sheep or baboons. Second, the studies differed in several parameters of the exposure and mostly they did not provide any information about the time of the year at which they were conducted.

In cattle, Burchard *et al.*^14^ reported contradictory results between two replicates, whereas other studies^15^,^16^ found no effect of ELFMF on MLT secretion in cows. No effect on the total day MLT but a significant difference in the day-night distribution was reported for mice^17^ and a slightly increased MLT secretion was found in adult rats^18^. In humans, short-term studies with volunteers exposed to artificial magnetic fields mainly failed to demonstrate an effect on MLT levels while studies on populations living in the vicinity of strong ELF electric as well as magnetic fields hinted towards a disruptive effect on MLT secretion^5^.

Recently, the MLT hypothesis was revisited, revised, and extended^1^,^2^. According to this modified hypothesis, ELFMF affects magnetoreception, which in turn disrupts circadian rhythmicity, MLT secretion, and affects vegetative physiology. The authors pointed out that thus far almost exclusively adult animals of only a limited number of species were tested, while, with respect to the suspected risk of childhood leukemia, especially juvenile animals should be studied. This view is supported by the few studies that compared ELFMF effects in younger and older rats, where effects where effects were always more pronounced in younger animals^19^,^20^. Also, it is mostly the long-term studies (>4 weeks exposure) that demonstrated partial inhibition of MLT secretion^6^,^8^, supporting the claim that ELFMF exposure should be long when addressing its possible effects.

In another line of evidence for biological effects of ELFMF, Burda *et al.*^21^ reported disturbed magnetic alignment of cattle grazing and resting under or near high voltage power lines. Magnetic alignment, a preference for a body orientation along the north south axis, is otherwise, in undisturbed areas, highly significant^22^,^23^,^24^. These findings constitute evidence for magnetic sensation as well as evidence of an overt behavioral reaction to weak ELFMFs in cattle, which, in principle, implies effects at the cellular and molecular levels.

These findings summarized above and our theoretical considerations about the importance of the study of juvenile animals led us to design and carry out a controlled experimental study of ELFMF exposure effects on salivary MLT levels in cattle calves. We exposed the calves to ELFMF at the intensity of 0.4 μT because this was regarded as the value where the risk of childhood leukemia is increased twofold in humans, as we think that this might be connected to the disturbance of the melatonin rhythm (cf.^18^).

## Results

**Figure 1:**
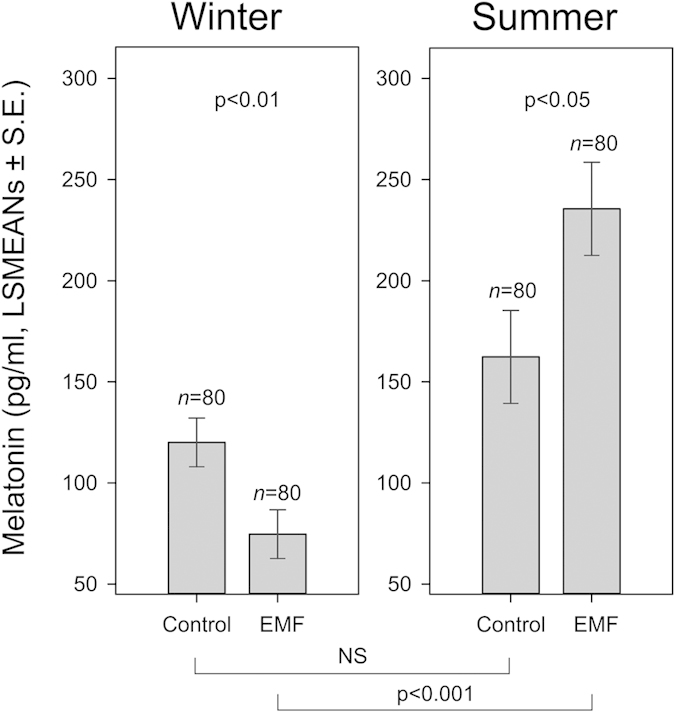
Melatonin concentrations (pg/ml) for control and experimental (EMF) calves (LSMEANs ± S. E.) in winter (left) and summer (right).

**Figure 2:**
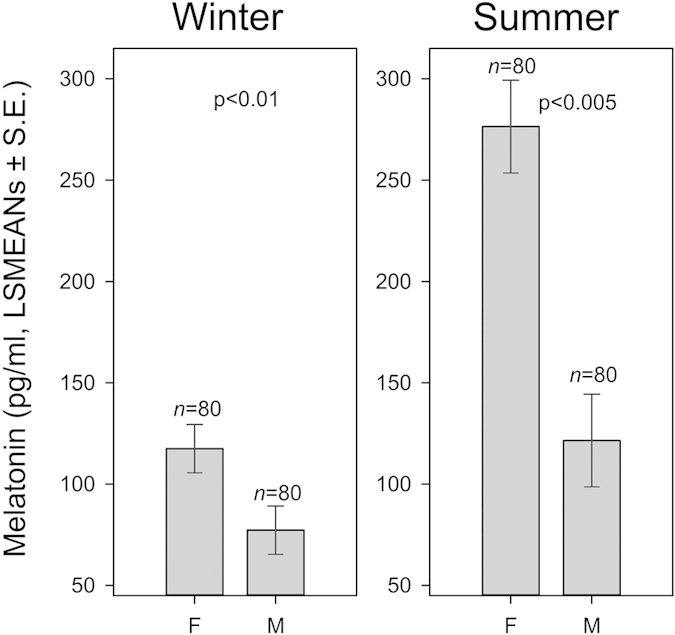
Melatonin concentrations (pg/ml) for female (F) and male (M) calves (LSMEANs ± S. E.), values from control and experimental animals combined, in winter (left) and summer (right).

**Figure 3:**
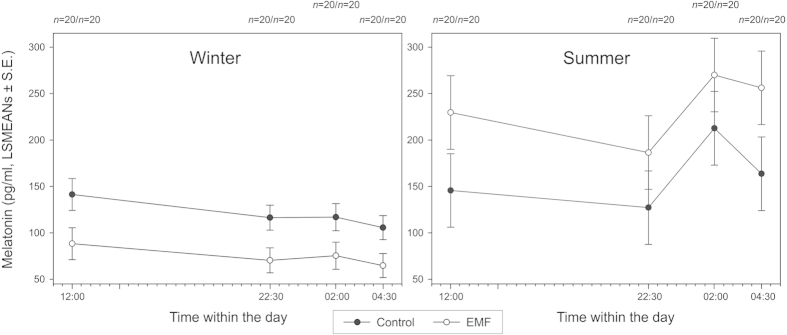
Melatonin concentrations (pg/ml) during the day for control and experimental (EMF) calves (LSMEANs ± S. E.) in winter (left) and summer (right).

**Figure 4:**
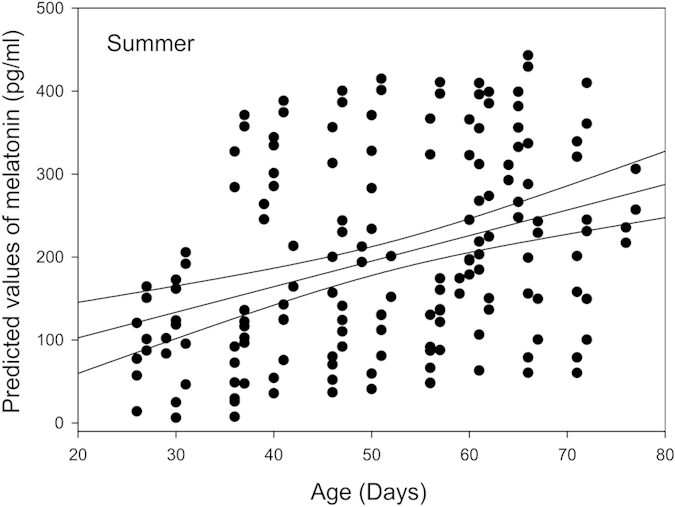
Predicted values of melatonin concentrations (pg/ml) in summer plotted against the age of the calves with a confidence interval (95%).

The results of the multivariate General Linear Mixed Model (GLMM) for the daily (averaged over 24 h) MLT concentrations in winter (GLMM1) revealed dependence on the treatment (i.e. control versus experimental) (F_1, 40 _= 7.54, p = 0.009, Fig. 1 left) and Sex (F_1, 40 _= 6.68, p = 0.014, Fig. 2 left). MLT concentrations were not affected either by the age of the calf, or its body weight. For design reasons (in order to get LSMEANs values), interaction between Time within the day and ID of the calf was kept in the model in spite of its non-significance (F_6, 49.3_ = 1.39, n.s., Fig. 3 left). In summer, MLT concentrations were dependent on Group (F_1, 40.5 _= 5.01, p = 0.031, Fig. 1 right), Sex (F_1, 40 _= 24.07, p < 0.001, Fig. 2 right), and Age (F_1, 40 _= 8.93, p = 0.005, Fig. 4). Also in this case, the interaction between Time within the day and ID of the calf was kept in the model although it was not significant (F_6, 96.1 _= 1.04, n.s., Fig. 3 right). MLT concentration was consistently higher in females than in males, both in control and experimental animals. For pooled data (GLMM3), MLT concentrations were dependent on the interaction between Group and Season (F_3, 80 _= 8.07, P < 0.001) and Sex (F_1, 80 _= 24.97, p < 0.001).

The average total daily MLT concentration in control animals in winter tended to be lower than in summer, but the difference was not significant (Fig. 1). The average total MLT concentration in the experimental animals compared to the control increased in summer and decreased in winter. The difference between the control and the respective experimental levels in each season was significant (GLMM1, t = 2.75, p = 0.009; GLMM2, t = −2.24, p = 0.03), as well as the difference between the winter and summer experimental levels (GLMM3, t = 4.79, p < 0.001, Fig. 1).

The MLT concentration in summer changed between the respective sampling periods and thus in the course of the day. The maximum MLT concentration was found in the 02:00 a.m. samples (Fig. 3). The daily course of changes was, particularly in winter, rather flat.

## Discussion

### Mean MLT saliva concentrations

The values of MLT saliva concentrations found in our study (mean 145, SD 101, range 33–400 pg/ml, n = altogether 40 daily values from altogether 8 control calves) were within the range of those reported for cattle in earlier studies. Since absolute values might be, among others (see below) also dependent on the applied methods, comparisons of absolute MLT concentrations determined in different studies should be done with caution. Comparison of values obtained under different conditions within a single particular study and determined with the same method is, however, fully appropriate.

### Daily course of changes, differences between winter and summer

In most studies, the season of the year in which the study had been performed was not specified. Usually the animals had been transferred to a closed stable and exposed to a certain controlled photoperiod for few days to few weeks prior to the experiment. However, it is well known that MLT synthesis in the pineal gland is strongly influenced by light. Accordingly, the MLT secretion shows marked daily rhythmicity with lower values during the day and higher concentration of MLT at night, regardless of whether the animals are diurnal or nocturnal. In general, the peak concentration is reached in about the middle of the dark phase. Since light stimulation is involved in the regulation of the MLT, the photoperiod is reflected in the production of MLT. The length of the secretion phase is negatively correlated with day length and thus longer in winter and shorter in summer (cf. i.a.^25^).

In pigs, characteristic MLT profiles with night maxima are maintained not only during natural day/night regime but also in constant darkness, however not under constant light conditions. Other authors^26^ , however, reported night maxima in pigs only under LD 12:12. The night MLT increase (in pigs) was found to be larger in winter but smaller in summer^27^ . The light intensity and light quality needed to suppress MLT production seem to be species specific, yet there is a large scatter in values given by different studies even for one and the same species. This brief and by far not complete survey shows that the characteristic text-book-like circadian MLT concentration profile with a clear night maximum need not be the rule in all species, and throughout the whole year (in different photoperiods). It should be tested in further studies how much the MLT concentration and its daily variation is really species-specific and how much the season of the year (photoperiod or natural circannual rhythm) influences the secretion rhythm.

### Influence of sex and age

The existing data on the influence of sex and age on MLT secretion in juvenile ungulates are contradictory. To the best of our knowledge there is no study of MLT concentration in cattle comparing males and females. An influence of sex and age was found between adult male and female pigs and between adult and juvenile male pigs but not between adult and juvenile females^27^. The highest MLT concentration in pigs was found between the 3rd–5th months^28^. MLT rhythms might be very weak at the ages of our calves (in reindeer “inexistent at the age of 15 days”)^29^. Valtonen *et al.*^30^, however, gives more marked rhythms (already in cattle calves aged 13–30 days) than those reported by us. Skrzypczak^31^ reported a significantly lower MLT levels in 2.5 months old calves when compared to cows. Also the overnight profile of the melatonin production was different in cows and calves; indicating that melatonin synthesis and release in the dark phase changes with age. Consequently, we have to be cautious to extrapolate our results to older individuals.

### Effect of ELFMF

In accordance with the melatonin hypothesis we found an inhibitory effect of ELFMF upon the secretion of MLT in winter, but, contrary to expectations, a stimulatory effect in summer. The inhibitory effect in winter was nevertheless much stronger than the positive effect in summer. These seemingly contradicting results find, however, some support also in existing publications, which have thus far not found much resonance in the community and were sorted in the general category of inconsistent, non-supporting, or contradictory results. For example, Jentsch *et al.*^32^ found a stimulatory effect of ELFMF during light phases in rats while in the dark phases the difference to controls was non-significant. The authors, however, did not interpret their findings. Löscher *et al.*^33^ found a 20% MLT increase in rats exposed to ELFMF in light and a 40% decrease in rats exposed during darkness. The MLT concentration varied also as a function of duration of exposure. An increase of rat MLT after ELFMF exposure was attributed to the length of exposure^18^. It should be noted at this point that light levels in the calf boxes were quite low at 20 lx. This is the level which in humans would see the rise in nocturnal melatonin^34^. The results presented here therefore apply only to the light exposure of the cattle in this experiment and may not be repeatable in other situations i.e. they could be a source of “failed replication” in future experiments, if not properly controlled for.

The mechanism behind these paradoxical (?) effects remains obscure. MLT synthesis in the pineal gland is controlled by the suprachiasmatic nucleus, and is regulated by several internal and external factors, of which light is the prominent one. While light inhibits the production of MLT, it stimulates the production of serotonin, precursor of MLT, and serotonin accumulates in the pineal during the light phase (cf.^35^). Conversely, the expression of serotonin N-acetyltransferase (SNAT, the enzyme which catalyzes the conversion of serotonin to MLT) is highest during the dark phase (cf.^35^). It was found that the SNAT activity in the pineal gland was suppressed by the exposure of animals to changing artificial magnetic field and (as a consequence?) serotonin levels were increased^35^,^36^. Alternatively, a changing magnetic field could directly stimulate the serotonin synthesis (cf.^35^). ELFMF might potentiate the effect of light (cf.^37^,^38^) and stimulate the serotonin synthesis during the light phase while it suppresses the synthesis of MLT during the dark phase. It might be of relevance that the depression effect of the changing magnetic field upon SNAT activity and MLT content was stronger during the dark period than during the light period^38^,^39^. However, even a scenario when ELFMF actually suppress the synthesis of serotonin but stimulates the synthesis of MLT should be tested. If so, there would be a large reserve of serotonin (due to stimulation by the long light period – which overrides the suppression by ELFMF) in the summer night and more MLT will be produced under exposure to ELFMF than without exposure. In winter, the reserve of serotonin will be small due to the short light period and suppression of its synthesis by ELFMF so that the reserve will be soon depleted and less MLT will be produced during the dark period.

**Figure 5:**
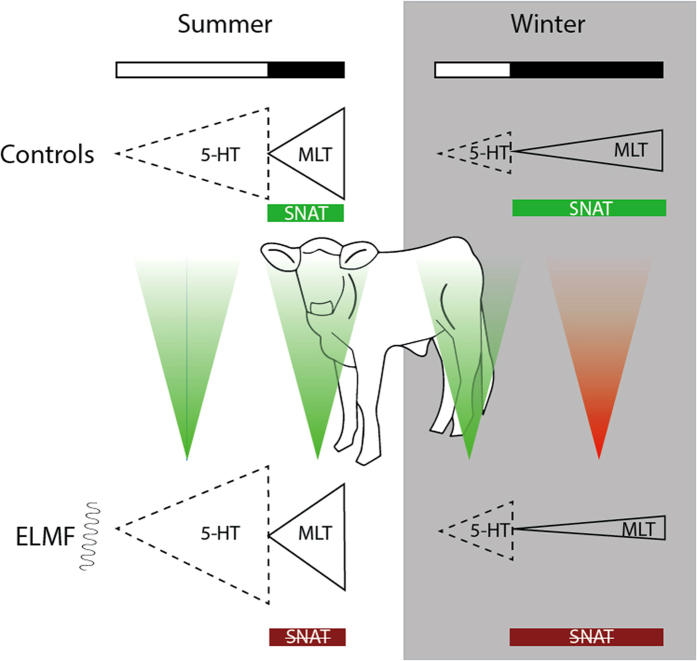
Possible explanation for the differential effect of ELFMF-exposure on MLT-levels in summer and winter. In the control calves (upper panel) the long photoperiod in summer leads to high amounts of accumulated serotonin (5-HT) at the end of the day when the serotonin N-acetyltransferase activity (SNAT, which is reduced through light exposure) starts to convert 5-HT into MLT. In winter, the short photoperiod prevents the synthesis of large 5-HT amounts and thus also caps MLT-levels. In the ELFMF-exposed calves (lower panel) the SNAT-activity is reduced at day- and nighttime. This leads to even higher 5-HT levels at daytime (not all of the 5-HT is converted to MLT during the night), especially during the long summer photoperiods (in addition, the 5-HT synthesis might be directly enhanced by ELFMF-exposure). In summer, this increased daily serotonin production overrides the effects of reduced SNAT activity at night, leading to higher MLT levels than in control calves. In winter, however, the photoperiod is too short for the 5-HT promoting effect of ELFMF to be of significance, leading to only a marginal increase in 5-HT. On the other hand, the longer dark period enhances the inhibiting effect of ELFMFs on SNAT activity, leading to lower MLT levels in exposed calves. Green indicates an increase or in the case of SNAT an active period. Red indicates a decrease or period of reduced activity, respectively. The dashed lines indicate that 5-HT levels were not measured in this study. (Author of the figure: E. P. Malkemper)

We suggest that in animals (species or individuals) which have a rather flat circadian profile of MLT secretion with a less pronounced maximum and minimum of the MLT concentration (as was the case in our calves), the ratio between the lengths of the light phase (during which serotonin accumulates) and the dark phase (during which serotonin is converged to MLT) and thus the amount of stored serotonin might compensate or overweight or potentiate the effect of ELFMF which depresses the activity of SNAT (Fig. 5).

Therefore, as also previously noted^39^, the possibility of an association between circadian rhythms and/or circannual rhythms, the photoperiod and/or the ambient temperature should be considered in future research when the effects of ELFMF are evaluated. Furthermore, the analysis of the ELFMF effects should focus also on activity of SNAT and the metabolism and concentration of serotonin.

While serotonin might be the effector of the effect of ELFMF on MLT production, the primary mechanism of possible interaction of ELFMF with the circadian clock remains unknown. According to the recent cryptochrome hypothesis^11^,^12^, ELFMF might exert a directly disturbing effect on circadian clock regulation. Taking into account the magnetosensitivity of ruminants (see above), one might otherwise consider the possibility of an indirect disrupting effect that would be mediated by magnetosensory stimulation (cf.^1^).

## Material and Methods

### Ethical note

The authors declare that the present study was carried out in accordance with the current laws of the Czech Republic and all the experimental protocols were approved by Animal Care and Use Committee of the Institute of Animal Science (VÚŽV 4/2013) and the Ministry of Agriculture of the Czech Republic (MZe 234439/2012-MZe-17214/2013).

### Study subjects

**Figure 6:**
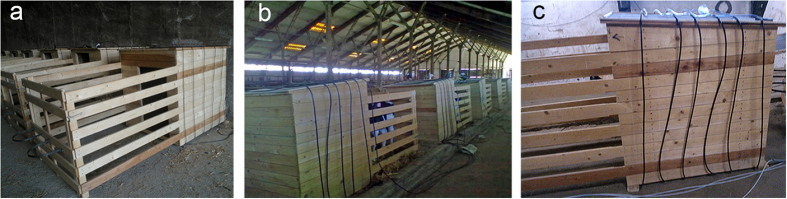
Individual wooden boxes for calves with coils producing ELFMF.

Two groups of eight calves (Holstein and Czech Red Pied cattle) of both sexes (1:1), aged 31.1 ± 1.5 days (mean ± SD) and weighing 61.3 ± 2.4 kg at the beginning of the experiment, and 114.7 ± 4.5 kg at the end of the experiment, were housed in individual wooden boxes for 35 days, one group in November/December 2013 (winter experiment) and another group in July/August 2014 (summer experiment) (Fig. 6).

### Location

The experiment was performed in the experimental stable of the Institute of Animal Science in Netluky (Southeast periphery of Prague, 50°02'22.94"N, 14°36'43.48"E).

### Nutrition

Calves were fed with cow milk twice a day (05.00 h a 17.00 h); granulated feed mixture and water were offered ad libitum.

### Light and temperature regime

Light intensity (illuminance) was measured by means of the luxmeter Extech HD450 (max. resolution 0.1 Lux). Light intensity in winter during the day (12.00 h) was around 30 lx in the open enclosure in front of the boxes (zero inside the boxes). Light intensity in summer (12.00 h) was about 85 lx in the enclosure and up to 20 lx inside the boxes. At night, the light intensity was always below the detection threshold of our luxmeter.

Artificial light (OSRAM Lumilux L 58W/840 fluorescent bulbs emitting only visible light, i.e. no UV light) in the stable was used only during the feeding time (05.00 and 17.00 h) for max. 1 h. A headlamp with dim red light was used during sampling.

The average ambient temperature (in the enclosure outside the boxes) was 2.7 °C during the winter experiment and 26.2 °C during the summer experiment.

### Exposure to ELFMF

Four calves (2 M, 2 F) from each experiment (winter and summer) were exposed to 50 Hz-MF (sine-wave, intensity 0.39–0.41 μT), produced by a custom built coil. The wooden boxes were oriented with its longer axis along 17° /197° and this was also the main direction of the oscillation of the artificial magnetic field. The local geomagnetic field intensity was 48.98 μT. The geomagnetic field inclination was 66° and essentially unchanged by the applied oscillating fields. The coils consisted of 6 turns of coaxial cable (shielded to prevent the generation of electrical fields) connected to a custom built power supply (Fig. 6). ELFMF intensities were measured at each sampling day with help of an Emdex Lite magnetic field meter (display rate: 4 seconds).

### Saliva sampling

The Sarstedt Salivette system was used to sample the saliva, a collection system which has already been successfully employed for hormonal analyses in cows^40^. Saliva collection using cotton buds and measurement of MLT in saliva offers a valid, non-invasive, pain-free and practical alternative to blood sampling and determination of serum MLT^40^. The test tube swab was applied into a calf’s mouth for at least 1 minute. The saliva soaked sponge was then put back to the test tube and stored in a styrofoam box on dry ice for transport to the laboratory. A clean pair of latex gloves was used for each individual sampling, to avoid sample contamination through transferred saliva of previous calves.

### Sampling regime

During the sampling period (35 days), saliva was sampled in regular intervals (sampling days 0, 10, 20, 30 and 35). Every sampling day involved four samples from each calf, collected at 12.00 noon, 10.30 PM, 02.00 AM and 04.30 AM.

### Transportation and storing

In each experiment (winter and summer) 8 × 4 × 5 = 160 samples were collected (320 samples altogether from both experiments). The samples were stored at −20 °C until analysis (not longer than 2 months).

### Sample preparation and analysis

MLT concentration in saliva samples was analyzed by ELISA (Enzyme-Linked ImmunoSorbent Assay - BlueGene Biotech., Bovine MLT Elisa Kit, catalogue number E11M0005). Before the analysis, the samples were thawed and centrifuged (3000 rpm for 15 minutes, 5 °C) to collect the saliva from the swab. Subsequently, the salivary samples were processed according to the ELISA kit instructions. The plates were read at 450 nm on a Thermomax plate reader (Molecular devices, USA), and data were analyzed by associated software (Softmax). The analysis was performed at the State Veterinary Institute Prague.

### Statistical Analysis

We tested the association between MLT concentrations and other variables (fixed and random effects) using a multivariate General Linear Mixed Model (GLMM, PROC MIXED, SAS System version 9.3, SAS Institute Inc.). As recommended by^41^, we generally followed several steps when using the MIXED procedure to analyze repeated measures data (see also^41^,^42^,^43^). First, we specified fixed effects. These were Group (Control / Experiment), time of day (12:00, 22:30, 02:00, and 04:30), Age of calf (22 to 69 days), Sex (Male / Female), Body weight at the beginning of the experiment (42 to 64 kg in winter, 62 to 64 kg in summer) and Body weight at the end of the experiment (102 to 145 kg in winter, 80 to 125 kg in summer). An interaction between Time within the day and ID of the calf was added to the model as a fixed factor. Data for winter and summer were analysed separately.

Second, we specified the covariance structure for “between subject” and “within subject” effects. In general, we assumed that repeated measures within a subject are correlated (SUBJECT = ID of a calf nested within the day of testing in the REPEATED statement), and the repeated measures between subjects are independent (Time within the day in the REPEATED statement). We used the TYPE = simple (VC), compound symmetric (CS), autoregressive (AR(1)), Toeplitz (TOEP), and unstructured (UN) option in the REPEATED statement to specify the covariance structure in each block. Third, following^41^, we compared candidate covariance models with various covariance structures. Based on Akaike’s information criterion AIC^44^ and Schwarz’s Bayesian criterion BIC^45^ we found that the unstructured covariance model with random effect for ID of a calf nested within the day is best fitting for winter and autoregressive covariance model with random effect for ID of a calf nested within the day is best fitting for summer data.

For each class we used least-squares-means (LSMEANs). LSMEANs are, in effect, within-group means appropriately adjusted for the other effects in the model.

## Additional Information

**How to cite this article**: Kolbabová, T. *et al.* Effect of exposure to extremely low frequency magnetic fields on melatonin levels in calves is seasonally dependent. *Sci. Rep.* **5**, 14206; doi: 10.1038/srep14206 (2015).

## References

[b1] Vanderstraeten J. & Burda H. Does magnetoreception mediate biological effects of power-frequency magnetic fields? Sci. Total Environm. 417-418, 299–304 (2012). 10.1016/j.scitotenv.2011.08.071 22071437

[b2] Vanderstraeten J., Verschaeve L., Burda H., Bouland C. & de Brouwer C. Health effects of extremely low-frequency magnetic fields: reconsidering the melatonin hypothesis in the light of current data on magnetoreception. J. Appl.Toxicol. 32, 952–958 (2012). 22696437 10.1002/jat.2761

[b3] Pall M. L. Electromagnetic fields act via activation of voltage-gated calcium channels to produce beneficial or adverse effects. J. Cell. Mol. Med. 17, 958–965 (2013). 23802593 10.1111/jcmm.12088 PMC3780531

[b4] Liboff A. R. Why are living things sensitive to weak magnetic fields? Electromagnetic Biology and Medicine, 33(3), 241–245 (2014). 23915203 10.3109/15368378.2013.809579

[b5] Henshaw D. L. & Reiter R. J. Do magnetic fields cause increased risk of childhood leukaemia via melatonin disruption? Bioelectromagnetics Suppl. 7, S86–S97 (2005). 10.1002/bem.20135 16059923

[b6] Jahandideh S., Abdolmaleki P. & Movahedi M. M. Comparing performances of logistic regression and neural networks for predicting melatonin excretion patterns in the rat exposed to ELF magnetic fields. Bioelectromagnetics 31, 164–171 (2010). 19771546 10.1002/bem.20541

[b7] Schüz J. Exposure to extremely low-frequency magnetic fields and the risk of childhood cancer: Update of the epidemiological evidence. Progr. Biophys. Molec. Biol. 107, 339–342 (2011). 10.1016/j.pbiomolbio.2011.09.008 21946043

[b8] Touitou Y. & Selmaoui B. The effects of extremely low-frequency magnetic fields on melatonin and cortisol, two marker rhythms of the circadian system, Dial. Clin. Neurosci. 14, 381–399 (2012). 10.31887/DCNS.2012.14.4/ytouitou PMC3553569 23393415

[b9] World Health Organization. Extremely Low Frequency Fields. Environmental Health Criteria Monograph no.238. WHO Press, Geneva (2007), http://www.who.int/peh-emf/publications/elf_ehc/en (Date of access: 01/02/2015).

[b10] Stevens R., Wilson B. & Anderson L . (Eds.) The Melatonin Hypothesis, Breast Cancer and Use of Electric Power. (Battelle Press, Columbus, 1997).

[b11] Lagroye I., Percherancier Y., Juutilainen J., Poulletier de Gannes F. & Veyret B. ELF magnetic fields: Animal studies, mechanisms of action. Progr. Biophys. Molec. Biol. 107, 369–373 (2011). 10.1016/j.pbiomolbio.2011.09.003 21914452

[b12] Maeda K. *et al.* Magnetically sensitive light-induced reactions in cryptochrome are consistent with its proposed role as a magnetoreceptor. Proc. Nat. Acad. Sci. USA 109, 4774–4779 (2012). 22421133 10.1073/pnas.1118959109 PMC3323948

[b13] Mirick D. K. & Davis S. Melatonin as a biomarker of circadian dysregulation. Cancer Epidemiol Biomarkers Prev. 17, 3306–3313 (2008). 19064543 10.1158/1055-9965.EPI-08-0605

[b14] Burchard J. F., Nguyen D. H., Monardes H. G. & Petitclerc D. Lack of effect of 10 kV/m 60 Hz electric field exposure on pregnant dairy heifer hormones. Bioelectromagnetics 25, 308–312 (2004). 15114640 10.1002/bem.20020

[b15] Burchard J. F., Nguyen D. H. & Monardes H. G. Exposure of pregnant dairy heifer to magnetic fields at 60 Hz and 30 microT. Bioelectromagnetics 28, 471–476 (2007). 17492762 10.1002/bem.20325

[b16] Rodriguez M., Petitclerc D., Burchard J. F., Nguyen D. H. & Block E. Blood melatonin and prolactin concentrations in dairy cows exposed to 60 Hz electric and magnetic fields during 8 h photoperiods. Bioelectromagnetics 25, 508–515 (2004). 15376244 10.1002/bem.20024

[b17] Kumlin T., Heikkinen P., Laitinen J. T. & Juutilainen J. Exposure to a 50-Hz magnetic field induces a circadian rhythm in 6-hydroxymelatonin sulfate excretion in mice. J. Radiat. Res. 46, 313–318 (2005). 16210787 10.1269/jrr.46.313

[b18] Dyche J., Anch A. M., Fogler K. A., Barnett D. W. & Thomas C. Effects of power frequency electromagnetic fields on melatonin and sleep in the rat. Emerg. Health Threats J. 5, 10904 (2012). 10.3402/ehtj.v5i0.10904 PMC3334267 22529876

[b19] Selmaoui B. & Touitou Y. Sinusoidal 50-Hz magnetic fields depress rat pineal NAT activity and serum melatonin. Role of duration and intensity of exposure. Life Sci. 57, 1351–1358 (1995). 7564882 10.1016/0024-3205(95)02092-w

[b20] Selmaoui B. & Touitou Y. Age-related differences in serum melatonin and pineal NAT activity and in the response of rat pineal to a 50-Hz magnetic field. Life Sci. 64, 2291–2297 (1999). 10374919 10.1016/s0024-3205(99)00180-0

[b21] Burda H., Begall S., Červený J., Neef J. & Němec P. Extremely low-frequency electromagnetic fields disrupt magnetic alignment of ruminants. Proc. Nat. Acad. Sci. USA, 106, 5708–5713 (2009). 19299504 10.1073/pnas.0811194106 PMC2667019

[b22] Begall S., Červený J., Neef J., Vojtěch O. & Burda H. Magnetic alignment in grazing and resting cattle and deer. Proc. Nat. Acad. Sci. USA, 105, 13451–13455 (2008). 18725629 10.1073/pnas.0803650105 PMC2533210

[b23] Begall S. *et al.* Further support for the alignment of cattle along magnetic field lines. Reply to Hert *et al*. J. Comp. Physiol. A. 197, 1127–1133 (2011). 10.1007/s00359-011-0674-1 PMC3221857 22028177

[b24] Slabý P., Tomanová K. & Vácha M. Cattle on pastures do align along the North-South axis, but the alignment depends on herd density. J. Comp. Physiol. A, 199, 695–701 (2013). 10.1007/s00359-013-0827-5 23700176

[b25] Wehr T. A. Melatonin and seasonal rhythms. J. Biol. Rhythms 12, 518–527 (1997). 9406025 10.1177/074873049701200605

[b26] McConnell S. J. & Ellendorf F. Absence of nocturnal plasma melatonin surge under long and short artificial photoperiods in domestic sow. J. Pineal Res. 4, 201–210 (1987). 3598855 10.1111/j.1600-079x.1987.tb00857.x

[b27] Green M. L., Clapper J. A., Andres C.J. & Diekman M.A. Serum concentrations of melatonin in prepubertal gilts exposed to either constant or stepwise biweekly alteration in scotophase. Dom. Anim. Endocrinol. 13, 307–323 (1996). 10.1016/0739-7240(96)00045-8 8839625

[b28] Anderson H. Plasma melatonin levels in relation to the light-dark cycle and parental background in domestic pigs. Acta Vet. Scand. 42, 287–294 (2001). 11503374 10.1186/1751-0147-42-287 PMC2202321

[b29] Eloranta E., Timirsjarvi J., Nieminen M., Ojutkangas V., Leppaluoto J. & Vakkuri O. Seasonal and daily patterns in melatonin secretion in female reindeer and their calves. Endocrinology 130, 1645–1652 (1992). 1537312 10.1210/endo.130.3.1537312

[b30] Valtonen M., Kangas A. P., Voutilainen M. & Eriksson L. Diurnal rhythm of melatonin in young calves and intake of melatonin in milk. Animal Sci. 77, 149–154 (2003).

[b31] Skrzypczak W. F. Circadian changes of the melatonin concentration in the blood of pregnant cows and calves. Acta Veter. Brno. 67, 153–158 (1998).

[b32] Jentsch A., Lehmann M., Schon E., Thoss F. & Zimmerman G. Weak magnetic fields change extinction of a conditioned reaction and daytime melatonin levels in the rat. Neurosci Lett. 157, 79–82 (1993). 8233036 10.1016/0304-3940(93)90647-4

[b33] Löscher W., Mevissen M. & Lerchl A. Exposure of female rats to a 100-μT 50 Hz magnetic field does not induce consistent changes in nocturnal levels of melatonin. Radiat. Res. 150, 557–567 (1998). 9806598

[b34] Zeitzer J. M., Dijk D.-J., Kronauer R. E., Brown E. N. & Czeisler C. A. Sensitivity of the human circadian pacemaker to nocturnal light: melatonin phase resetting and suppression J. Physiol. 526.3, 695—702 (2000). 10922269 10.1111/j.1469-7793.2000.00695.x PMC2270041

[b35] Lerchl A., Nonaka K. O., Stokkan K. A. & Reiter R. J. Marked rapid alterations in nocturnal pineal serotonin metabolism in mice and rats exposed to weak intermittent magnetic fields. Biochem. Biophys. Res. Commun. 169, 102–108 (1990). 1693500 10.1016/0006-291x(90)91439-y

[b36] Welker H. A. *et al.* Effects of an artificial magnetic field on serotonin N-acetyltransferase activity and melatonin content of the rat pineal gland. Exp. Brain Res. 50, 426–432 (1983). 6641877 10.1007/BF00239209

[b37] Sarrias M. J., Artigas F., Martínez E. & Gelpí E. Seasonal changes of plasma serotonin and related parameters: Correlation with environmental measures. Biol. Psychiat. 26, 695–706 (1989). 2478199 10.1016/0006-3223(89)90104-2

[b38] Lambert G. W., Reid C., Kaye D. M., Jennings G. L. & Esler M. D. Effect of sunlight and season on serotonin turnover in the brain. Lancet 360, 1840–1842 (2002). 12480364 10.1016/s0140-6736(02)11737-5

[b39] Burchard J. F., Nguyen D. H. & Block E. Effects of electric and magnetic fields on nocturnal melatonin concentrations in dairy cows. J. Dairy Sci. 81, 722–727 (1998). 9565875 10.3168/jds.S0022-0302(98)75628-0

[b40] Stärk K. D. C. *et al.* Absence of chronic effect of exposure to short wave radio broadcast signal on salivary melatonin concentrations in dairy cattle. J. Pineal Res. 22, 171–176 (1997). 9247202 10.1111/j.1600-079x.1997.tb00320.x

[b41] Littell R. C., Pendergast J. & Natajan R. Modeling covariance structure in the analysis of repeated measures data. Statistics in Med. 19, 1793–1819 (2000). 10.1002/1097-0258(20000715)19:13<1793::aid-sim482>3.0.co;2-q 10861779

[b42] Verbeke G. & Molenberghs G. Linear Mixed Models for Longitudinal Data. (Springer Verlag, 2000).

[b43] Tao J., Littell R., Patetta M., Truxillo C. & Wolfinger R. Mixed Model Analyses Using the SAS System Course Notes. (SAS Institute Inc., 2002).

[b44] Akaike H. A new look at the statistical model identification. IEEE Transaction on Automatic Control AC 19, 716–723 (1974).

[b45] Schwarz G. Estimating the dimension of a model. Ann. Statist. 6, 461–464 (1978).

